# Can positive expectations help to improve the learning of risk literacy? A cluster-randomized study in undergraduate medical students

**DOI:** 10.1186/s12909-022-03498-1

**Published:** 2022-05-31

**Authors:** Sven Benson, Katharina Schmidt, Julian Kleine-Borgmann, Stephanie Herbstreit, Manfred Schedlowski, Anke Hollinderbäumer

**Affiliations:** 1 Institute of Medical Psychology and Behavioral Immunobiology, Center for Translational Neuro- and Behavioral Sciences, University Hospital Essen, University of Duisburg-Essen, 45147 Essen, Germany; 2 Institute of Medical Education, Center for Translational Neuro- and Behavioral Sciences, University Hospital Essen, University of Duisburg-Essen, 45147 Essen, Germany; 3 Department of Neurology, Center for Translational Neuro- and Behavioral Sciences, University Hospital Essen, University of Duisburg-Essen, Essen, Germany; 4 Department of Orthopedics and Trauma Surgery, University Hospital Essen, University of Duisburg-Essen, Essen, Germany; 5 Department of Clinical Neuroscience, Osher Center for Integrative Medicine, Karolinska Institutet, 171 77 Stockholm, Sweden; 6 Institute of Medical Biostatistics, Epidemiology and Informatics (IMBEI), University Medical Centre, University of Mainz, Mainz, Germany

**Keywords:** Framing, Expectation, Placebo, Risk communication, Risk literacy, Statistics anxiety, Learning barriers

## Abstract

**Background:**

Risk literacy, i.e., the ability to calculate and apply risk parameters, represents a key competence for risk communication and medical decision making. However, risk literacy is reportedly low in medical students. The successful acquisition of statistical competencies is often difficult, and can be hampered by emotional learning obstacles, calling for interventions to support learning. In this cluster-randomized study, we aimed to translate findings from placebo research to medical education. Specifically, we tested if the acquisition of risk literacy during a seminar unit can be facilitated by positive expectations, induced by a positive and non-threatening framing of the content and learning goals.

**Methods:**

The study took place during a mandatory 2.5-h seminar on “risk literacy” for 2^nd^ year medical students. The seminar teaches both statistical knowledge and its application in patient communication. To test the effects of expectations on risk literacy acquisition, the (otherwise identical) seminar was framed either as “communication training” (positive framing condition) or “statistics seminar” (negative framing condition). All *N* = 200 students of the semester were invited to participate, and cluster-randomized to the positive or negative framing condition (4 seminar groups each condition). Risk literacy was assessed with the “Quick Risk Test” (QRT) at the beginning and end of the seminar, along with statistics anxiety and subjective learning success using questionnaires.

**Results:**

Data from *N* = 192 students were included. At the end of the seminar, risk literacy was increased in both framing conditions, with a significantly greater increase in QRT scores in the positive framing condition. Statistics anxiety was significantly decreased in both framing conditions, with no evidence of group differences. Subjective learning success was overall high and comparable between groups.

**Conclusions:**

Supporting our hypothesis, positive framing led to a significantly greater increase in risk literacy (i.e., in QRT scores). Our data offer first support that positive framing of learning goals may help to facilitate the acquisition of statistical knowledge. Expectation-orientated interventions may thus offer a feasible tool to optimize learning settings and framing of learning objectives in medical statistics courses.

## Background

For health care professionals, a fundamental understanding of statistical analysis and parameters is crucial for the critical reading of scientific publications and medical decision making [1, 2]. In addition, the competence to correctly apply, calculate and interpret statistical parameters is an important prerequisite for medical communication and patient counseling. This is particularly true for risk parameters, which represent a statistical basis for risk communication and medical decision making [3, 4]. However, risk literacy, defined as the ability to correctly calculate and apply risk parameters such sensitivity, specificity, or positive predictive value, is reportedly low in medical students and even in medical residents [3–6]. This may have negative consequences for patient care, e.g. if patients are not correctly informed about treatment options [3, 5]. Thus, educational interventions have been designed to improve risk literacy in medical students, which have overall promising effects [7–10]. These teaching units primarily aim at cognitive learning objectives such as the acquisition of knowledge about risk parameters and the use of solution strategies. It is likely that deficits in risk literacy are not solely caused by cognitive or strategical problems [11], but also by emotional learning barriers [11]. Indeed, the acquisition of statistical competencies represents a major stress and anxiety-inducing topic in undergraduate and graduate students across various disciplines [12, 13]. Emotional learning barriers like negative emotions towards statistics (i.e., “statistics anxiety”) or a negative mathematical self-concept can reportedly interfere with the successful acquisition and application of statistical knowledge [12, 14–16], with potentially debilitating effects on the performance in statistics courses ([13]; for critical review see [17]).

Against this background, the question arises how the acquisition of risk literacy can be facilitated, and how emotional learning barriers be reduced. One promising approach may derive from placebo research: Increasing evidence supports that positive expectations, typically related to a sham treatment (e.g., with placebo pills), can improve the performance in various cognitive tasks. Beneficial expectation effects have been demonstrated for different higher-order cognitive functions such as memory [18, 19], fluid intelligence [20], general knowledge [21], and creativity [22]. Recently, a randomized controlled trial in medical students demonstrated positive effects of a 3-week open-label (i.e., non-deceptive) treatment with placebo pills on emotional well-being during preparation for a mid-term exam [23], indicating that positive expectations can counteract stress and negative affectivity in the context of learning in medical students. It is thus intriguing to hypothesize that an expectation-based intervention could offer a novel approach to improve the acquisition of medical statistics, either directly or indirectly by reducing statistics anxiety as an emotional learning barrier. While most placebo studies used “vehicles” such as placebo pills, we herein adopted the concept of framing, which allows to induce positive expectations by verbal information alone. A different framing of identical information (e.g., positive framing “60 of 100 lives saved” versus negative framing “40 of 100 lives lost”) affects information processing and decision making, as demonstrated by Tversky & Kahneman [24]. Framing has already been utilized to induce positive and to reduce negative treatment-related expectations in patients [25]. Until yet, it has not been investigated if framing could also help to create a supportive learning environment.

Thus, we herein aimed to assess if positive framing leads to an improved acquisition of medical statistical knowledge, i.e. risk literacy. We further explored if positive framing could reduce statistics anxiety as an emotional learning barrier, or increase the subjective relevance of the topic, which might modulate the putative effect of framing on learning outcome. Insights from this study might help to improve our understanding how learning objectives should be worded to optimize the effects of risk literacy courses (e.g., [7, 8]).

## Methods

### Study aim and hypotheses

Aim of this cluster-randomized study was to test if the acquisition of risk literacy can be improved by positive framing. Different verbal information regarding the learning goals were used to frame an established seminar on risk literacy either as “communication training” (positive framing) or as “statistics seminar” (negative framing). The "statistics seminar" framing focused on the need to acquire statistical knowledge to understand scientific reports, corresponding to our usual procedure. The positive framing was designed to induce positive expectations and to reduce emotional learning barriers which might hamper the acquisition of risk literacy. To this end, framing directed the participants´ attention to the communication-related learning aims of the seminar, i.e., the training of communication skills needed for risk communication and medical decision making. Of note, the seminar had a similar content in both conditions, and comprised statistical knowledge and its application in patient communication, making both framing conditions truthful and convincing for students. We specifically hypothesized that framing as "communication training" when compared to “statistics seminar” leads to a greater increase in risk literacy, assessed with the standardized Quick Risk Test [8]. Further, we expected that positive framing would reduce statistics anxiety, and increase subjectively-rated learning success and perceived relevance of the topic (secondary outcomes). Finally, we aimed to explore putative moderating effects of learning barriers on risk literacy acquisition.

### Study design and setting

This cluster-randomized study was conducted between October 2019 and February 2020 (winter term 2019/2020) at the Medical Faculty of the University Duisburg-Essen, Germany. The study took place during the course “Medical Psychology and Medical Sociology”, which constitutes a mandatory course for 2^nd^ year medical students. A total of *N* = 200 students participated in the course during winter term 2019/2020, in 8 consecutive groups of approximately *N* = 25 students. The 2.5-h seminar on “risk literacy” is an established, integral part of the course, and constitutes the first course on risk literacy or risk communication in the curriculum at our faculty. To test the effects of framing in a naturalistic classroom setting, seminar groups were randomized cluster-wise (group-wise) to the "communication training" or the "statistics seminar” framing condition, respectively (see below). Risk literacy (primary outcome) and secondary outcomes were assessed at the beginning (baseline, before randomization and framing intervention took place) and at the end of the seminar using the same set of standardized questionnaires (see below).

### Ethical considerations and confidentiality

All seminar groups were informed that we aimed to assess how the didactic approach of the seminar can be improved. Students were invited to answer the questionnaires, and were informed in writing that study participation was voluntary, i.e., that they were free to answer or to return unfilled questionnaires. Data were collected anonymously. Participants were asked to create an individual code based on recommendations of the German Society for Psychology (DGPs), which allows to combine the repeatedly measured questionnaire data (at the beginning and end of the seminar) without the possibility to identify study participants. The study was approved by the Institutional Ethics Review Board and the Data Protection Officer of the Medical Faculty of the University of Duisburg-Essen (permit number: 19–8976-BO).

### Power calculation and study sample

A priori calculation of sample size revealed that a sample size of *N* = 186 participants was necessary to detect small-sized effect (f = 0.12) with a statistical power of 1-β = 0.90 (α = 0.05) in ANOVA time x group interaction effects for change in QRT scores. Thus, all *N* = 200 2^nd^ year medical students of the university of Duisburg-Essen who participated in the mandatory course during winter term 2019/2020 were considered as target sample (i.e., the full semester group) and invited to take part in the study. Data from students who returned fully completed QRT questionnaires (i.e., primary outcome) at the beginning and end of the seminar (*N* = 192, see results section) were included into analysis. The high number of participants supports that the study sample is representative for the target sample, with a low risk of sampling bias (e.g., non-response or participation bias, respectively, of students with a high or low affinity to statistics). Since the seminar is the first course on risk literacy or risk communication in the curriculum, participants had little to no pre-existing clinical knowledge or experience on this topic.

### Randomization

To test effects of framing in this naturalistic classroom setting, cluster randomization was used, i.e., entire seminar groups were randomly assigned to the “communication training" or the "statistics seminar” framing condition (i.e., each four out of the eight seminar groups to one of the framing conditions). Cluster randomized studies are an appropriate form of randomized controlled trials if a randomization of individual study participants is not possible, e.g. when educational measures are assessed in a classroom setting [26, 27]. All seminars were given by the same lecturer (i.e., by the first author of the study). To preclude the lecturer from influencing the assignment of respective seminar groups to a study condition, sealed and numbered envelopes which contained the respective framing condition were pre-prepared by an independent researcher, and consecutively opened by the lecturer in the seminar room, immediately before the framing intervention started.

### Framing

The framing intervention was conducted at the beginning of the risk literacy seminar, according to randomization information. In both framing conditions, the framing intervention comprised highly standardized (see below) scripted verbal and written information related to the background, major topics and learning goals of the seminar. Two presentations with identical slides were prepared, except one slide containing the framing-related content (see below). Of note, the seminar combined the training of statistical and communication-related skills, which allowed to give truthful information in both framing conditions.

#### Positive framing condition

The positive framing aimed to induce positive expectations and to reduce emotional learning barriers which could interfere with the acquisition of risk literacy. To this end, the lecturer primarily emphasized the communication-related topics of the seminar, and put the statistical content in the background. Participants were informed that the overarching goal of the seminar was to improve communication skills in the context of risk communication and medical decision making. Students were also told that they would learn basic statistical tools and practical competencies for patient counselling. This would allow to explain risk parameters to patients and lay persons in a simple and an easy understandable way. The exact wording was (key messages were additionally shown in the presentation): “In the following, we will talk about risk communication. In your professional life, you will very often make decisions together with your patients, for example about different therapy options, or explain the meaning of a test result. This means that you need to inform and counsel your patients accordingly. Risk scores are an important tool for informing patients in a comprehensible and comprehensive way. Thus, the goal of the seminar is to achieve an understanding of basic statistical tools for risk communication, and achieve the competency to communicate them in a simple and easy understandable way”.

#### Negative framing condition

In the negative framing condition, the need to acquire statistical knowledge for a good understanding of scientific literature was emphasized. In detail, participants were informed that the primary learning goal was to improve statistical knowledge and skills, which are important to read and understand scientific medical literature. They would thus learn important basic statistical concepts and approaches to calculate risk parameters. The reference to patient communication was only mentioned in passing. The exact wording was (key messages were additionally shown in the presentation): “In the following, we will talk about risk parameters. Knowledge about risk parameters is important for the correct understanding of statistical findings and scientific articles, which provides the basis for evidence-based medicine. Thus, the goal of this seminar is to achieve an understanding of basic statistical concepts and to achieve the competence to correctly calculate and interpret risk parameters.”

In both framing conditions, students were informed that the medical statistical concepts would be tested as part of the exam. All information was provided in a standardized manner, using written and scripted verbal information. In addition, framing information was repeated at various time points during the seminar, with a comparable number of “reminders” in both study conditions (e.g. “helpful tool for patient communication”; “this is needed to inform patients in an easy understandable way” versus “this statistical knowledge is helpful when reading scientific literature”, “this is needed to understand statistical information”).

### Didactic conception of the risk literacy seminar

The risk literacy seminar constitutes an established and integral part of the compulsory course “Medical Psychology and Medical Sociology”, which has been developed and delivered for more than five years by the first author of the study (S.B.). Briefly, the seminar consists of a brief knowledge-based introduction of risk parameters (i.e., sensitivity, specificity, positive and negative predictive values, influence of prevalence), small group exercises to calculate risk parameters using natural frequency trees, a role play related to medical decision making (communication of positive predictive values), and group discussions. Importantly, the seminar was conducted in a standardized manner and its content did not differ between both framing conditions. Nevertheless, differences related to the interaction with the respective seminar groups could not be fully excluded in this real-life classroom setting.

### Outcome measures

Outcome measures were assessed with standardized questionnaires at the beginning and at the end of the risk literacy seminar.

#### Risk literacy (primary outcome)

Risk literacy was assessed with the standardized Quick Risk Test (QRT; 8). The QRT was constructed to evaluate basic medical statistical concepts, which are specifically related to screening tests. The QRT is sensitive to change and has been used to test the efficacy of teaching interventions in pre-post designs [8]. In the original publication by Jenny et al. [8], final year students had a pretest median of 53.8% (interquartile range, IQR = 44.4% – 68.5%) of correct answers, and showed a significant improvement after a 90-min training session performance to a median of 92.3% (IQR = 83.2% – 94.2%). In the pretest, median item discrimination index was 0.23 (IQR = 0.14 – 0.28), indicating a good item discrimination between students with higher and lower total scores. We herein used the first seven items to assess the understanding of sensitivity, specificity, positive predictive value (PPV), negative predictive value (NPV), prevalence, Bayesian reasoning, and risk reduction. Three remaining items of the 10-item QRT were discarded since the therein tested content was not covered by the seminar. All QRT items have a multiple choice format with four possible and one correct answer. Herein, the number of correct answers was summarized as an indicator of risk literacy, with scores ranging from 0 to 7 and higher scores indicating more correctly answered questions.

#### Mathematical self-concept and statistics anxiety (predictor variables)

Academic mathematical self-concept was assessed with two items, adapted from [28]. Participants were asked to rate how good they are in mathematics and how they performed in previous mathematics courses on seven-point Likert ranging from “very poorly” to “very well”. According to [28], the two items show a high internal consistency (Cronbach´s alpha = 0.88) and can be aggregated for analysis. Herein, a sum score was calculated, with lower scores indicating a more negative mathematical self-concept. Statistics anxiety as an emotional learning barrier was measured with four four-point Likert items, ranging from “does not apply” – “applies in full”. Items were derived from the 17-item “Worry, Avoidance, and Emotionality Cognitions Encountering Statistical Demands (WAESTA)” scale [12]. Reliability (Cronbach´s alpha = 0.92–0.94) as well as internal and external validity of the WAESTA scale were considered as adequate [12]. For the purpose of this study, four items were selected to address 1) worries regarding the ability to meet statistics requirements in medical studies, 2) difficulties to understand statistical content in a seminar, 3) worries “to make a fool of myself” if one had to comment on statistical data in a seminar, and 4) feeling uncomfortable if one has to work on a statistical problem. Corrected item-test correlation for these items ranged from r_it_ = 0.44–0.69. Mathematical self-concept and statistics anxiety were assessed at the beginning of the seminar as putative predictors of learning success. To additionally explore changes in statistics anxiety as a secondary outcome, statistics anxiety items were also assessed at the end of the seminar.

#### Subjective relevance of the topic and subjective learning success (secondary outcomes)

The subjective relevance of the topic, i.e. to acquire statistical knowledge and skills, was assessed at the beginning and at the end the seminar with seven self-constructed five-point Likert-scaled items ranging from “fully applies” to “does not apply at all” (unpublished survey, University Medical Center Mainz, Germany). In detail, personal interest in acquiring statistical knowledge, the perceived importance of acquiring statistical knowledge in medical studies and the perceived general importance of statistical knowledge for the medical profession were addressed, together with items on the subjective importance of acquiring statistical knowledge during medical studies in relation to doctor-patient communication, clinical-practical (e.g., internal medicine, ophthalmology) and clinical-theoretical topics (e.g., microbiology, pharmacy), respectively.

Subjective learning success was measured at the end of the seminar with two self-constructed 5-point Likert-scaled items (ranging from “fully applies” to “does not apply at all”) addressing how well students felt prepared for the upcoming exam and for the calculation of risk parameters in general. For analyses, sum scores were used, with higher scores indicating higher subjective success.

### Statistical methods

Framing condition groups were compared with Chi^2^-test and independent sample t-tests regarding sociodemographic and baseline psychological characteristics. To assess changes in primary and secondary outcome parameters and potential differences between study conditions, repeated measures analyses of variance (rmANOVA) with the within-subject factor time and the between-subject factor group were calculated. For detailed analyses of significant interaction effects, posthoc independent sample t-tests (with Bonferroni-correction where appropriate) were computed. In addition, two stepwise multiple linear regression analyses were computed across the full sample to explore associations between QRT test scores and with putative predictor variables (according to 17). The first regression analysis aimed to test associations between the change in QRT scores (i.e., delta for QRT scores assessed at the beginning and the end of the seminar) as dependent variable and framing condition, change in statistics anxiety (i.e., delta beginning to end of seminar), mathematical self-concept, sex, age, and advanced courses in mathematics as predictor variables. The second regression analysis explored predictors of absolute QRT test scores (assessed at the end of the seminar), with framing condition, baseline statistics anxiety, mathematical self-concept, sex, age, and advanced courses in mathematics as predictors. All data are shown as mean ± standard deviation, if not otherwise indicated. Data analyses were performed with IBM SPSS statistics, version 27 (IBM Corp., Armonk, New York, USA).

## Results

### Study participants

A total of *N* = 192 students were included in the study (i.e., 96% of all 200 students who attended the seminar). Participants had a mean age of 21.7 ± 3.6 years (range 18–38 years), 68.6% were female and 31.4% male, and 38.0% had visited advanced courses in mathematics (“Leistungskurs”) during high school. Approximately one quarter of the participants (26.1%) had completed a vocational training before studying medicine. The groups did not differ in sociodemographic characteristics (Table 1; all *p* > 0.05). Mathematical self-concept and statistics anxiety at baseline were comparable between groups (Table 1; *p* > 0.05).

**Table 1:** Sociodemographic and baseline characteristics

	**“statistic framing”** (*N* = 97)	**“communication framing”** (*N* = 95)
Age (Mean ± SD)	21.6 ± 3.5	21.7 ± 3.7
Female % (N)	71.1 (69)	66.0 (62)
Advanced courses in mathematics (Leistungskurs Mathematik) % (N)	33.0 (31)	43.3 (39)
Vocational training % (N)	27.9 (27)	24.2 (23)
Subjective relevance (Mean ± SD)	16.1 ± 4.7	16.4 ± 4.6
Mathematical self-concept baseline (Mean ± SD)	10.6 ± 2.8	10.8 ± 2.2
Statistics anxiety baseline (Mean ± SD)	9.4 ± 2.9	9.1 ± 2.7

### Primary outcome: risk literacy

**Fig. 1:**
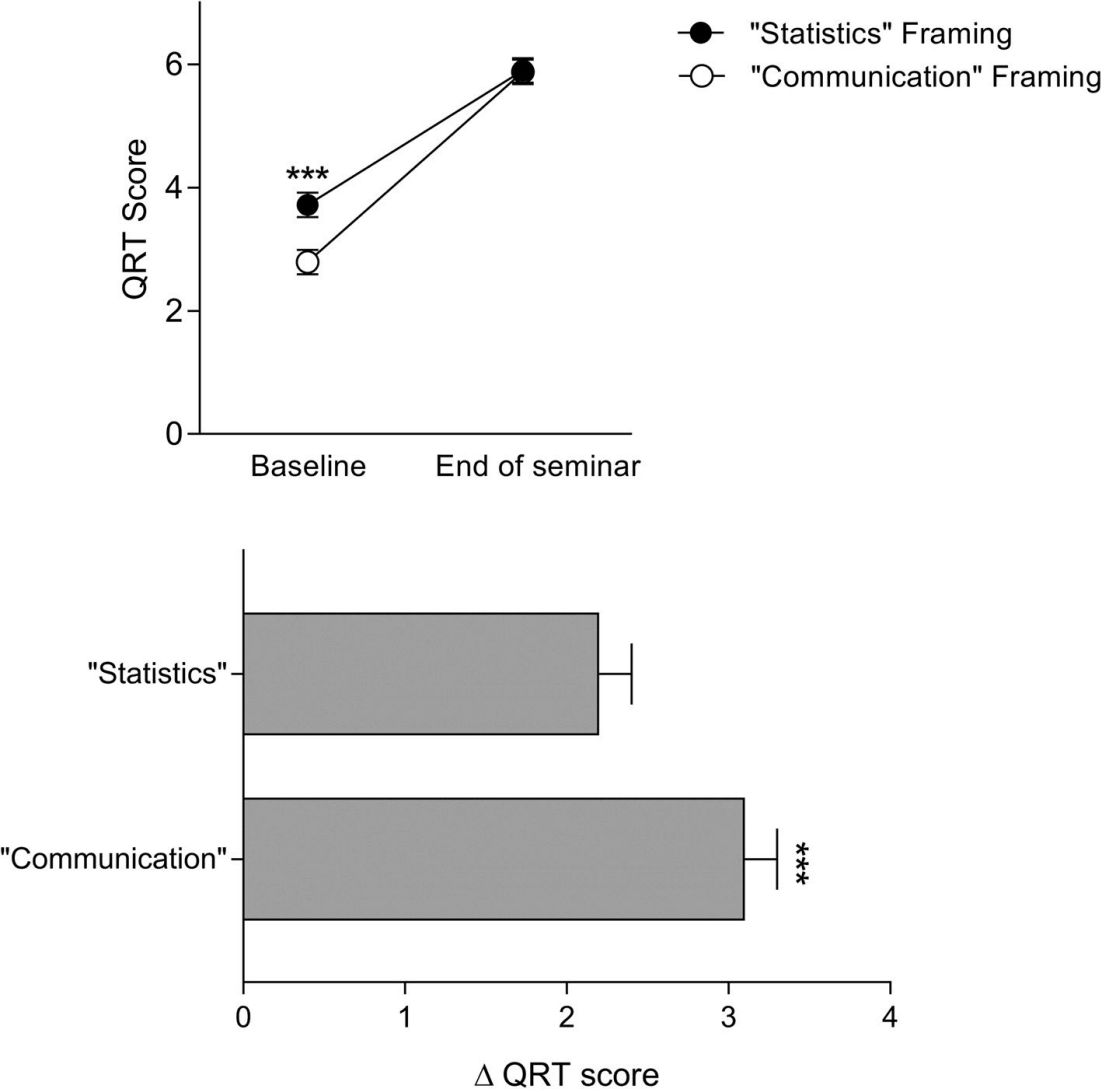
Quick Risk Test (QRT) scores assessed at the beginning (Baseline) and the end of the seminar (upper panel) as well as changes in QRT scores (delta scores; lower panel) in groups who received positive versus negative framing. ***p < .001, results of posthoc t-tests. For ANOVA results, see text. Data are shown as mean and SEM

A significant increase in risk literacy, i.e., in the number of correctly answered QRT questions, was observable in both framing condition groups (F_1;190)_ = 452.1; *p* < 0.001; ƞ_p_^2^ = 0.70), with a more pronounced increase in the communication framing condition (interaction time x group, F_(1;190)_ = 13.8; *p* < 0.001; ƞ_p_^2^ = 0.07). In detail, participants in the communication framing condition showed a significantly greater increase in QRT scores when compared to participants of the statistic framing (delta scores: 3.1 ± 1.9 versus 2.2 ± 1.5; t_(190)_ = -3.7, *p* < 0.001; Fig. 1, lower panel). Of note, framing condition groups showed a significant baseline difference, with lower scores in the communication framing group at the beginning of the seminar (t_(190)_ = 4.4, *p* < 0.001), while the number of correct answers was comparable at the end of the seminar (t_(190)_ = 0.1, *p* = 0.94) (Fig. 1, upper panel).

### Secondary outcomes: Statistics anxiety, subjective relevance and subjective learning success

**Fig. 2:**
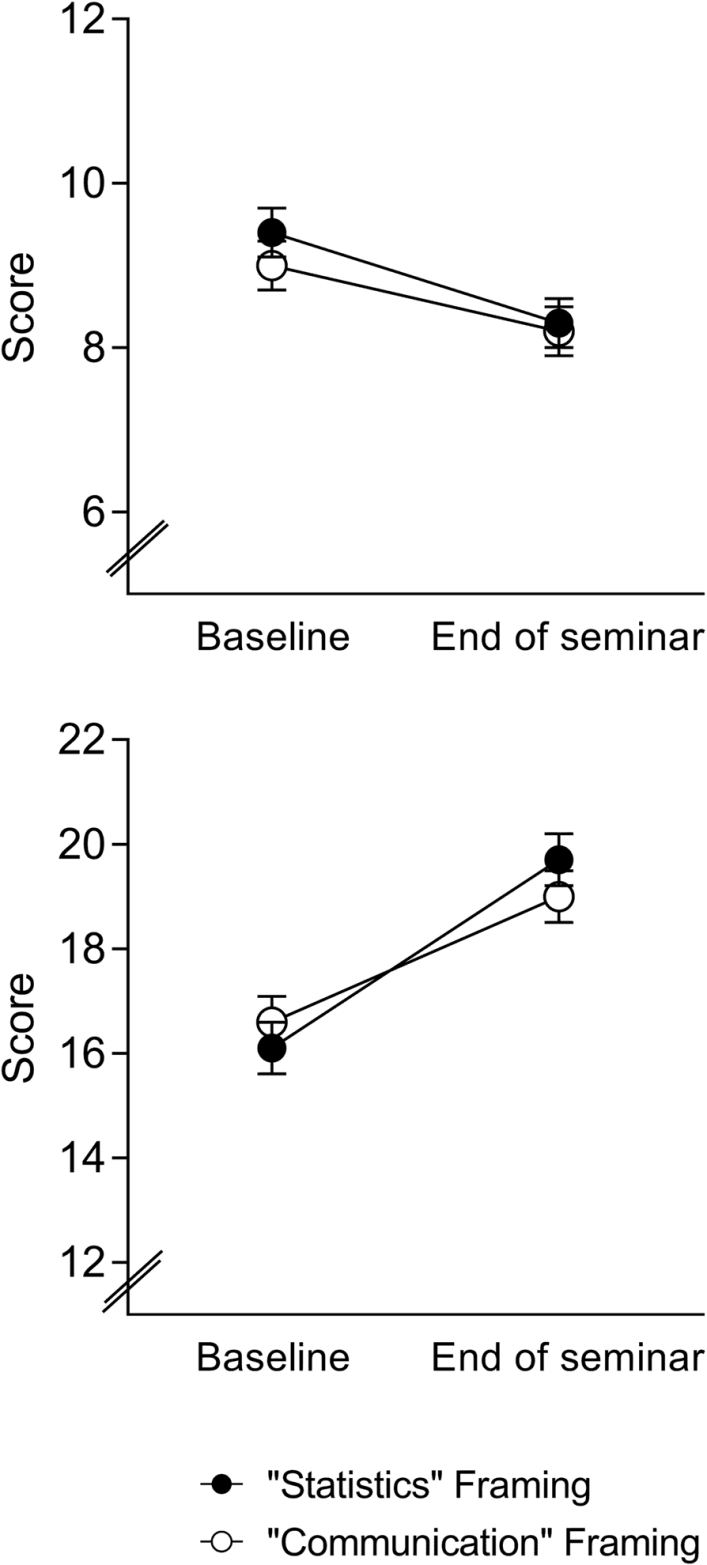
Statistics anxiety (upper panel) and subjective relevance of the topic (i.e., learning statistics in medical studies; lower panel) were assessed at the beginning (Baseline) and the end of the seminar. No differences were observed between groups who received positive versus negative framing. For ANOVA results, see text. Data are shown as mean and SEM

Both framing condition groups showed a significant decrease in statistics anxiety from the beginning to the end of the seminar (F_(1;190)_ = 26.9, *p* < 0.001, ƞ_p_^2^ = 0.14; Fig. 2, upper panel), with no evidence of group or time x group interaction effects.

The subjective relevance of the topic (i.e., to learn statistical knowledge and skills) was rated significantly higher at the end of the seminar as compared to the beginning (F_(1;175)_ = 92.6, *p* < 0.001, ƞ_p_^2^ = 0.35), with no evidence for group differences or a time x group interaction (Fig. 2, lower panel).

Subjective learning success, assessed at the end of the seminar, was overall high and comparable between groups (statistic framing: 8.9 ± 1.1; communication framing: 8.8 ± 1.1; t_(189)_ = 0.6, *p* = 0.55).

### Regression analyses

To explore predictor variables which may account for the variance in risk literacy, we conducted multiple regression analyses across all participants. For the increase in risk literacy (i.e., change in QRT scores from baseline to post seminar), framing group emerged as only significant predictor variable (b = 1.00, β = 0.28, t = 3.8, *p* < 0.001; model: F = 14.5, *p* < 0.001, adj. *R*^*2*^ = 0.07). This is in line with ANOVA results and indicates that positive framing is associated with a greater increase in risk literacy. No additional variance was explained by change in statistics anxiety, mathematical self-concept, advanced courses in mathematics, or sociodemographic variables. The absolute QRT score, assessed at the end of the seminar, was negatively correlated with statistics anxiety (b = -0.09, β = -0.24, t = -3.2, *p* = 0.001; model: F = 10.6, *p* = 0.001, adj. *R*^*2*^ = 0.05), indicating that participants who reported lower statistics anxiety at baseline achieved higher QRT scores after the seminar. This effect was independent of other predictor variables including framing condition.

## Discussion

Statistical literacy is reportedly low among medical students [8]. Thus, courses dedicated to teach a correct understanding and application of statistical parameters have been developed and implemented as key components of many medical curricula [7–10]. However, the successful acquisition of statistical knowledge and competencies is often difficult for students, and can be hampered by emotional learning obstacles such as statistics anxiety [11, 13]. In this cluster-randomized study, we tested if positive framing of the content and learning goals facilitates the acquisition of risk literacy in a seminar unit. In line with our hypothesis, we found that positive framing led to a significantly greater increase in risk literacy as assessed with the standardized QRT test. The beneficial effect of positive framing on risk literacy acquisition was also supported by multiple regression analysis, which accounted for other factors with potential influence on QRT performance. These data offer first support that a positive framing of learning goals may help to facilitate the acquisition of statistical knowledge. Our finding complements and extends an increasing body of evidence that positive expectations can enhance the performance in cognitive tasks [18–22, 29]. To induce positive expectations, these studies typically implemented the deceptive application of inert substances such as placebo pills, e.g. by suggesting that the pill contains a pharmacologically active substance such as caffeine. Since such procedures cannot be translated to teaching settings for both ethical and practical reasons, we herein utilized for the first time the concept of framing, which allows to induce positive expectations in a non-deceptive and practically well-realizable way.

We chose to test framing effects in the specific context of medical statistics, where learning barriers such as statistics anxiety have been shown to hamper the acquisition and application of statistical knowledge [11, 14, 15]. Indeed, our exploratory regression analysis revealed an association between higher baseline statistics anxiety and lower QRT scores (i.e., lower risk literacy) at the end of the seminar, which further supports the role of statistics anxiety as learning barrier [13]. Other factors such as the visit of advanced courses in mathematics which might reflect “an affinity” to statistics did not contribute to variance in regression models. As the seminar was conducted in 2^nd^ year students who had no previous courses in risk literacy or risk communication, effects of different pre-existing knowledge or clinical experience can be most likely excluded. It is possible that the debilitating effect of statistics anxiety on learning and performance can be explained by disrupted central executive processes, resulting in reduced working memory capacity [30] and / or decreased attentional control [31]. Against this background, our positive framing intervention aimed to reduce statistics anxiety as a putative learning barrier. To this end, we used a “non-threatening” framing in the positive condition which was not primarily related to the statistical contents of the seminar. Instead, we emphasized the acquisition of communication skills and practical competencies for patient counseling as main learning goals. Notably, the seminar comprised both statistic- and communication-related content, allowing a convincing and truthful framing of both framing conditions. However, against our expectation, we found that the decrease in statistics anxiety was comparable in both framing conditions. Moreover, although regression analysis indicated a beneficial effect of positive framing on risk literacy acquisition, we did not find evidence that this effect was associated with changes in statistics anxiety. This raises the question how the observed framing effect can be otherwise explained. It is well-suitable that positive framing may contribute to increased motivation or cognitive effort, and ultimately to harness unused cognitive resources, as previously suggested for placebo effects on cognitive performance [18, 21]. It is also possible that inhibitory mechanisms, which interfere with optimal performance, are reduced by positive expectations [21, 22]. Finally, positive expectations may have increased self-efficacy, which in turn leads to improved performance in cognitive tasks [32].

Independent of the framing condition, participants showed a significantly enhanced QRT performance, and reported a high subjective learning success at the end of the seminar. This finding in 2^nd^ year students with no specific pre-existing knowledge from prior curricular courses lends further support that the acquisition of risk literacy and the application of statistical knowledge can be effectively improved by a brief teaching intervention in medical students [7–9]. A recent prospective observational study showed that even a workshop of 90-min duration was suitable to increase risk literacy in final year medical students [8]. While this workshop was primarily designed to convey knowledge and strategies, others have combined knowledge-transfer with the practical training of risk communication skills [7, 9]. Similar to our seminar, Han and colleagues [7] used role play to improve risk communication skill in addition to knowledge transfer. Their three-hour program, evaluated in a small sample of second year medical students, led to significant increases both in subjective and objective measures of risk literacy [7], and was later successfully adopted as online course [33]. The existing studies, all conducted to analyze the effects of seminars or trainings on risk literacy acquisition, have either used multiple choice tests [8] or objective structured clinical examination (OSCE) [7, 9] as primary outcome. Herein, we implemented the standardized QRT according [8], aiming to test the effect of framing on risk literacy acquisition as primary outcome. The QRT was specifically developed as a change sensitive instrument for repeated assessments, allowing to test risk literacy in an objective and economic way. In future studies, it would be of interest to analyze the effects of framing also with regard to risk communication competencies, e.g. with OSCE.

### Strength and limitations

First of all, the framing condition groups showed significantly different increases (i.e., delta scores), but not absolute differences in QRT scores at the end of the seminar. This may partially be attributed to the significant baseline differences in QRT scores. Higher baseline scores in the statistics framing condition may have reduced the probability to show as large increases as in the communication framing group. An alternate explanation is that a considerable number of participants in both groups had reached the maximum in QRT scores at the end of the seminar. Thus, ceiling effects could have precluded a further increase. It is also possible that the performance in complex academic tasks can be influenced only in a limited range by expectation effects. For example, a recent randomized controlled trial in medical students showed that positive expectations, induced by an open-label (i.e., non-deceptive) placebo treatment, did not affect absolute performance in an academic test, while emotional well-being was improved [23]. Along the same lines, a laboratory placebo study found a significant expectation effect on the subjectively perceived, but not on the objectively measured cognitive performance [34]. We herein included 2^nd^ year students without specific pre-existing clinical knowledge or experience from medical studies. It remains an interesting question if the framing intervention would have a different effect in students with greater knowledge and / or experience. Further, our framing intervention was tested in a naturalistic class room setting, which provides ecologically valid results, but does not allow a strict standardization of the seminar. Thus, we cannot fully exclude that our findings were influenced by differences in the interaction with the respective seminar groups. However, both framing conditions were conducted in four seminar groups each to increase reliability, and the seminar followed a well-established structured didactic concept to minimize variability between groups. Moreover, the framing intervention was conducted by a researcher involved in this study, which may be a source of bias, either consciously or unconsciously (e.g., Rosenthal effect). To reduce bias risk, the framing intervention was strictly standardized with respect to verbal / written information to ensure structural equivalence [26], and the seminar itself was based on a structured didactic concept and standardized as far as possible (see above). From a methodological standpoint, it would be a more robust approach if framing and delivery of the course would be conducted by independent persons, allowing a blinding for the study condition. However, such a procedure could hardly be implemented in teaching practice and might reduce external validity, because framing and teaching would be conducted by the same person under “real life” conditions. Finally, we were only able to test the immediate effects of the intervention on risk literacy, but not the longevity of effects. Thus, future research is needed to replicate our findings—also with respect to other statistical competencies—in independent samples of students with different levels of clinical knowledge and experience, with teachers who are not involved in the study, aiming to further disentangle the underlying cognitive and emotional processes.

### Conclusion and practical implications for medical education

Our study amends previous findings that even short seminars are capable to increase risk literacy, and may have positive effects on emotional learning barriers and subjectively experienced relevance of medical statistics in undergraduate medical students. Our primary research aim was to test the effect of positive framing on the acquisition of risk literacy. Supporting our hypothesis, data indicate that framing as an expectation-orientated intervention can offer an innovative and practically feasible tool to optimize learning settings, especially in medical statistics courses. From a practical point of view, framing can be easily utilized when learning objectives are formulated. A positive framing of learning objectives may help to show and transmit enthusiasm for the subject being taught and thus to support a positive attitude to the learning subjects [35]. This might be of special importance in introductory courses or lectures, assuming that positive framing can help to encourage students to gain an autonomous motivation. According to self-determination theory [36], autonomous when compared to extrinsic motivation has positive effects on understanding, performance, and feelings of competence [35]. As a consequence, findings from placebo research including framing effects could be a relevant topic for didactic trainings. Importantly, herein it should be emphasized that expectation-oriented interventions should be positive in a realistic, but not over-exaggerated way to avoid a so-called expectation violation which easily results in disappointment or feeling of frustration [37].

## Data Availability

The dataset generated during the current study will not be shared publicly to guarantee privacy of participants, given that we cannot fully exclude that individuals could be identified. However, the data set will be available from the corresponding author on reasonable request.
